# Assessing the effectiveness of integrated watershed management practices and suggesting innovative strategies in southern Ethiopia

**DOI:** 10.1016/j.heliyon.2024.e38619

**Published:** 2024-09-28

**Authors:** Amare Tadesse Muche, Yohannes Smeneh Ketsela, Belete Meketaw Ali

**Affiliations:** Faculty of Water Resources and Irrigation Engineering, Arba Minch University, Water Technology Institute, Arba Minch, Ethiopia

**Keywords:** *Alternative management strategies*, *Community perceptions on conservation practices*, *Effectiveness of IWM practices*, *Land use land cover change*, *Soil water conservation*

## Abstract

Integrated watershed management plays a vital role in promoting sustainable water resource management and addressing environmental challenges. This study aims to analyze and assess the effectiveness of existing IWM practices and develop new strategies to improve watershed management. The data collection process encompassed comprehensive field observations, surveys, and consultations with the stakeholders. According to a hydrometer test, loam soil was the average dominant soil type in Elgo and Kola shell kebele. The assessment of existing soil water conservation initiatives adhered to the rigorous standards set by the Ministry of Agriculture. From 2016 to 2022, Elgo Kebele saw significant land use changes: agriculture expanded by 11.24 %, bare land by 2.05 %, water bodies by 1.79 %, and settlements by 0.54 %, while forests declined by 15.34 %. In Kola Shele, agriculture, water bodies, and settlements slightly increased by 0.5 %, 1.03 %, and 0.033 %, respectively, with decreases in bare land (1.82 %) and forest (0.05 %). Only 25 % of sampled plots met the criteria for effective soil water conservation systems, indicating challenges in current practices. For cultivated land with less than a 15 % slope and vertisol, recommended conservation practices include broad bed and furrow, conservation tillage, grass strips, grassland improvement, and mulching. For slopes greater than 50 %, hillside terracing, graded bunds, and trenches are advised. Additional measures, such as water harvesting, grass waterways, revegetation, and actions against illegal farming, were proposed. In summary, this study highlights the urgent need for improved IWM practices, and used to enhance watershed management, address environmental and socio-economic issues, and promote sustainable land use in the study.

## Highlights

• Assessment of Existing Practices: A comprehensive analysis of current IWM practices was conducted, identifying gaps and areas for improvement.
• Data Collection Methodology: The study utilized extensive field observations, surveys, and stakeholder consultations to gather relevant data on the study areas.
• Soil Type Analysis: Findings revealed that loam soil was the predominant type in Elgo and Kola Shele kebeles, informing effective soil water conservation strategies.
• Land Use Changes: Significant shifts in land use from 2016 to 2022 were documented, including an 11.24 % increase in agriculture in Elgo Kebele and a 15.34 % decline in forest cover.
• Effectiveness of Soil Water Conservation: Only 25 % of sampled plots met effective soil water conservation criteria, indicating substantial challenges in current practices.
• Recommended Conservation Practices: Tailored conservation strategies were proposed based on land slope, including broad bed and furrow, hillside terracing, and graded bunds.
• Proposed Additional Measures: The study advocates for supplementary actions such as water harvesting, grass waterways, and regulations against illegal farming to enhance watershed management.
• Call for Urgent Improvements: The study highlights the immediate need for enhanced IWM practices to improve watershed management, tackle environmental and socio-economic issues, and promote sustainable land use.

## Graphical abstract

**Figure:**
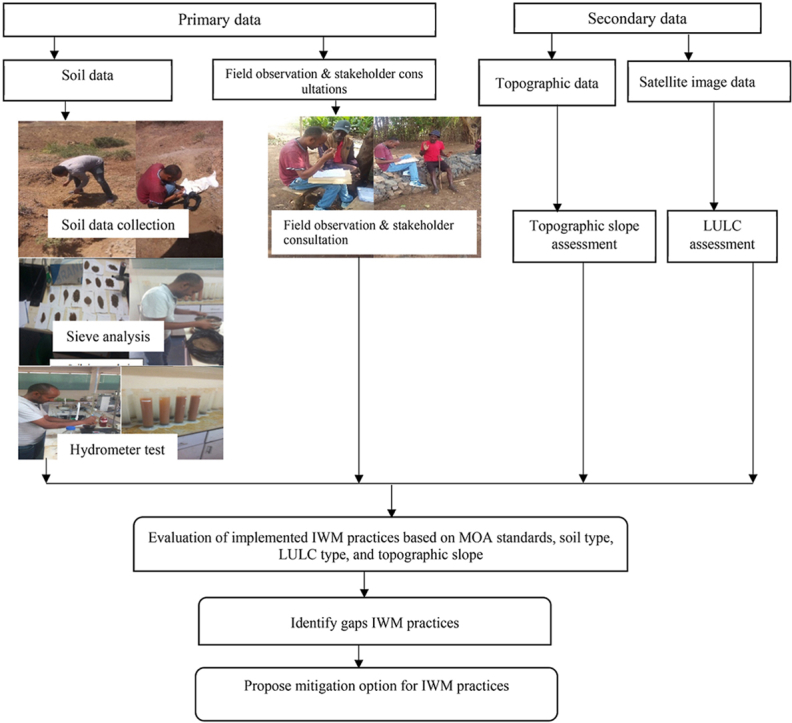

## 1 Introduction

Integrated Watershed Management (IWM) is a comprehensive strategy that effectively manages the complex relationships between land, water, and the environment within a watershed [1]. This approach aims to balance ecological sustainability with socio-economic development, ensuring the environment's and local communities' long-term well-being. With watersheds encountering escalating pressures from factors like population expansion, climate variability, and unsustainable land use practices, the need for assessment becomes increasingly urgent [2,3]. Evaluating the efficacy of current IWM practices and crafting innovative strategies are essential steps toward securing the enduring vitality and resilience of critical ecosystems [4]. The main aim of watershed management is to optimize the utilization of land, water, vegetation, and the environment, thereby mitigating flood risks, curbing soil erosion, and fostering sustainable production of various essential commodities such as food, fuel, livestock feed, fiber, and timber, ensuring long-term viability [5,6]. As emphasized in Ref. [7], The IWM strategy highlights the significance of involving communities in the sustainable management of land and water resources. Through a participatory approach, IWM seeks to enhance water and land productivity while upholding institutional and ecological integrity [7,8]. Consequently, watershed management has emerged as a pivotal facet of rural development and poverty alleviation agendas [9].

IWM helps restore degraded lands, enhances soil fertility, optimizes water resource use, increases agricultural output and non-farm activities, broadens income opportunities, and improves market access, providing advantages to both households and communities [10,11]. Moreover, it serves to broaden income opportunities and streamline market access. In essence, the dividends of IWM are palpably experienced by households and communities alike. Evaluating the efficacy and consequences of IWM practices is paramount for gauging their effectiveness and pinpointing avenues for enhancement. This assessment encompasses scrutinizing the outcomes of such practices concerning water availability, quality, biodiversity preservation, livelihood enhancement, and socio-economic progress [12].

Residents of cultivated watersheds in Ethiopia face critical threats to their livelihoods. To prevent further land degradation, there is an urgent need for the development of effective policies and strategies [13]. Achieving this objective requires precise scientific planning and execution, drawing on the technical expertise in watershed management practices and their impact on sustaining resources over the long term. IWM emerges as a viable solution to counteract land degradation [14]. As explained in the Government of Ethiopia's National Seminar on Watershed Management proceedings (2000), IWM entails the harmonization of technologies within the geographical confines of the drainage area, aimed at optimizing the utilization and development of land, water, and forest resources to meet the fundamental needs of the populace sustainably [15]. This approach mandates the integration of disparate initiatives such as soil conservation, afforestation, water resource development and management, and other rural development endeavors into meticulously planned micro watershed projects. Furthermore, the successful implementation of agriculture, forestry, and ecological restoration initiatives hinges upon resource management programs executed on a watershed basis, serving as a pivotal component of sustainable development [16].

Southern Ethiopia is characterized by a diverse landscape that includes highland plateaus and lowland plains, with a climate that varies significantly across the region [6]. These geographical and climatic differences result in complex hydrological patterns and distinct ecological zones. The downstream areas of the Sego watershed, particularly Elgo and Kola Shelle kebeles, are particularly vulnerable to flood risks from the highlands. The study area features highlands in the upper regions and lowlands in the lower regions. This topographical arrangement, rapid farmland expansion, deforestation, and frequent flooding, exacerbate the region's environmental challenges. Therefore, effective watershed management is critical to addressing these issues. The diverse topography of Southern Ethiopia influences water flow and distribution, leading to varied ecological and hydrological conditions within the region [17]. The highlands act as water catchment areas, where rainwater collects and flows down to the lowlands. This natural water flow is disrupted by human activities such as the extensive expansion of agricultural land and deforestation [18]. Removing vegetation for farming reduces the land's ability to absorb and retain water, increasing surface runoff and the risk of flooding downstream.

In the Elgo and Kola Shelle kebeles, the consequences of these disruptions are particularly severe. The highland areas receive substantial rainfall, which, without adequate vegetation cover, quickly flows down to the lowlands, carrying soil and causing erosion. This not only reduces soil fertility in the highlands but also leads to sedimentation and flooding in the lowlands, threatening local infrastructure and livelihoods [19]. The excessive flooding observed in these areas highlights the urgent need for comprehensive watershed management practices.

Proper watershed management involves a series of integrated practices designed to maintain the ecological balance while supporting sustainable land use. This includes reforestation, soil and water conservation techniques, controlled agricultural expansion, and the construction of flood control structures. By implementing these practices, the region can mitigate the adverse effects of flooding, reduce soil erosion, and enhance water conservation. To mitigate the risks in the region, the Kebele administration, in collaboration with the local community, has implemented various conservation practices such as soil bunds, stone bunds, stone-faced bunds, and gabion structures. While these measures have been beneficial, they are insufficient to fully address the challenges posed by flooding and land degradation. Consequently, it is essential to evaluate the effectiveness of the existing conservation structures and develop more comprehensive mitigation strategies. This study introduces a novel approach to IWM by evaluating and enhancing existing practices through comprehensive field observations, stakeholder consultations, and rigorous soil and land use analysis. Therefore, this study aims to evaluate the effectiveness of IWM practices and propose innovative Strategies for the Lower Sego Watershed in Southern Ethiopia.

## 2 Materials and methods

### 2.1 Study area description

**Fig. 1:**
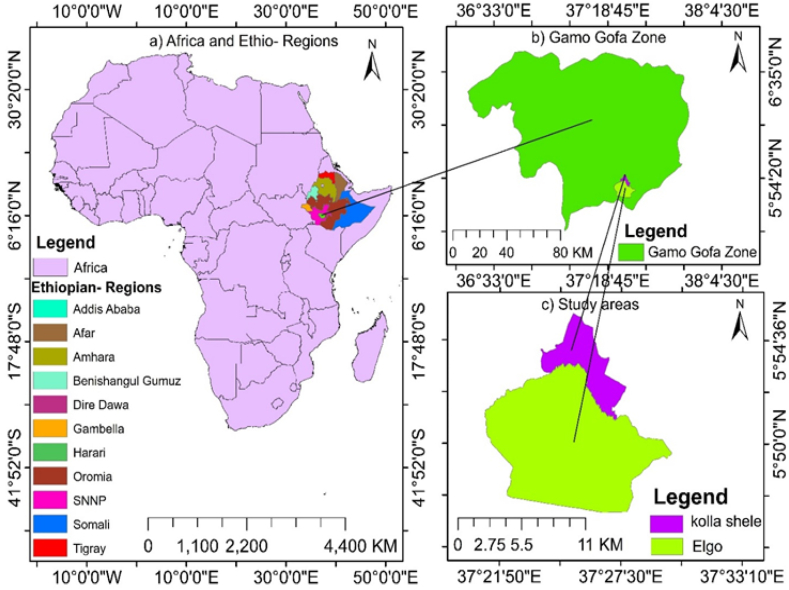
Location map of the study area.

The study is found under the Sego watershed which is located in Arba Minch zuriya woreda, Gamo Zone, in Southern Ethiopia. It is located at an average altitude of 1190m.a.s.l with a latitude of 5.48^0^ to 5.55^0^ in Northing and a longitude of 37.22^0^ to 37.29^0^ in Easting. The location map of the study area is shown in Fig. 1 below.

### 2.2 Climate condition

**Fig. 2:**
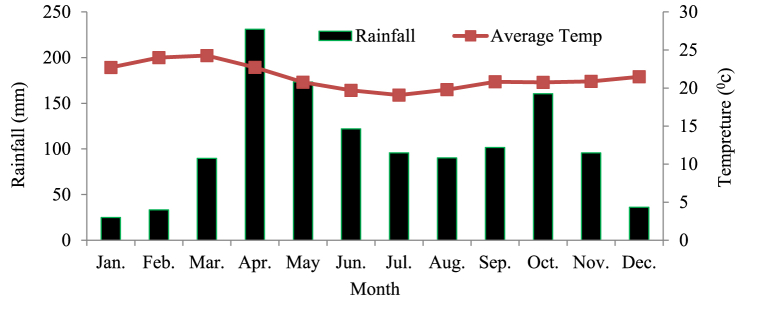
Monthly Rainfall(mm) and average temperature (^O^_C_).

The study area typically receives around 990mm of mean annual rainfall and shows a pattern where precipitation occurs in two distinct peaks throughout the year. The average annual temperature ranges from 14.4^0^_C_ to 32.4^0^_C_, based on data from the Geresse meteorological station. The mean annual rainfall and temperature are shown in Fig. 2 as follows.

### 2.3 Data collection methods

The primary data was collected through surveys and questionnaires. Collected data from local communities, farmers, and stakeholders to understand their perceptions and experiences with current IWM practices, interviews: conduct structured and semi-structured interviews with key informants such as local leaders, agricultural experts, and government officials, field observations: Observe the physical conditions of the study area, land use patterns, and implementation of management practices, and Focus Group Discussions (FGDs): engage with groups of stakeholders to gather in-depth insights and diverse perspectives. Soil data: the necessary soil physical properties were tested in the laboratory.

The secondary data collected from the literature review: review of existing research, reports, and case studies related to IWM in Southern Ethiopia, Remote Sensing, and GIS Data: use of satellite imagery and GIS data to analyze land use changes, vegetation cover, watershed conditions over time, and meteorological data: collect data on rainfall, river flow, soil moisture, and other relevant parameters from meteorological stations.

### 2.4 Land use land cover analysis

LULC change involves examining historical and present changes in LULC and comparing them to evaluate the extent and rate of natural resource degradation [20]. Sentinel-2 remote sensing was used to download the land use and land cover. The data span from 2016 to 2022 was selected to observe the changes over time. Sentinel-2 data was downloaded from the Copernicus Open Access Hub (https://scihub.copernicus.eu/). It provides high-resolution imagery with a spatial resolution of 10 m for visible and near-infrared bands, and 20 m for the shortwave infrared bands.

### 2.5 Land use land cover accuracy assessment

LULC accuracy assessment is a critical process to validate the accuracy of LULC maps produced using remote sensing and GIS technologies [21]. The assessment involves comparing the map's classification against ground truth data to determine the map's accuracy. Here are the steps used in LULC accuracy assessment. Data Collection: reference data corresponding to the LULC classes in the map. This involves ground-truthing to collect sample points that represent different LULC classes. Generate Confusion Matrix: Use the collected data to create a confusion matrix, known as a contingency table. Calculate Accuracy Metrics: Calculate accuracy metrics such as overall accuracy, user's accuracy, and kappa coefficient using the confusion matrix. Interpret the accuracy metrics to understand the reliability and limitations of the LULC map. High accuracy metrics indicate a high-quality map, while lower values suggest the need for refinement or additional data collection. The assessment is represented by (Eqs. (1), (2), (3))), which expresses the degree of compatibility between the classification and the reality as mentioned by Ref. [22].

1. Overall accuracy

(1) $$\text{Overall}\;\text{accuracy}=\frac{\text{Total}\;\text{number}\;\text{of}\;\text{correctly}\;\text{classified}\;\text{pixels}\left(\text{Diagonal}\right)}{\text{Total}\;\text{number}\;\text{of}\;\text{reference}\;\text{pixels}}*100$$

2. Users Accuracy

(2) $$\text{User}\;\text{accuracy}=\frac{\text{Number}\;\text{of}\;\text{correctly}\;\text{classified}\;\text{pixels}\;\text{in}\;\text{each}\;\text{catagory}}{\text{Total}\;\text{number}\;\text{of}\;\text{classified}\;\text{pixels}\;\text{in}\;\text{that}\;\text{category}\left(\;\text{Row}\;\text{total}\right)}*100$$

3. Kappa coefficient (Ḱ)

(3) $$\left(Ḱ\right)=\frac{N\left(\sum_{i=1}^{r}\text{ii}\right)-\left(\sum_{i=1}^{r}\left(\text{Xi}+-.X+i\right)\right)}{N^{2}-\sum_{i=1}^{r}\left(\text{Xi}+.X+1\right)}$$

The level of agreement and percentage of reliability data of Kappa is presented in Table 1 below as suggested by Ref. [22].

**Table 1:** Level of agreement and percentage of reliability data of Kappa.

Values of Kappa	Level of Agreement	% of reliable data
0–0.2	None	0–4%
0.21-0.39	minimal	4–15 %
0.4-0.59	weak	15–35 %
0.6-0.79	moderate	35–63 %
0.8-0.9	Strong	64–81 %
Above 0.9	Almost perfect	82–100 %

### 2.6 Soil conservation methods

Terracing involves constructing stepped terraces on slopes to minimize soil erosion and surface runoff. It is crucial for reducing water runoff, preventing soil erosion, and expanding arable land in hilly areas [23]. Contour plowing consists of plowing along the contour lines of a hill to create furrows that run perpendicular to the slope, which helps slow water runoff, enhance water infiltration, and reduce soil erosion. Agroforestry incorporates trees and shrubs into farming systems and agricultural landscapes [24]. These trees serve as windbreaks, reduce soil erosion, improve soil fertility through leaf litter, and provide additional resources like fruits and timber [25]. Crop rotation entails rotating different crops in the same field over multiple seasons. This method improves soil structure and fertility and reduces erosion [26]. Stone bunds involve placed of stones along contour lines to slow down water flow, reduce soil erosion, and trap sediments, thereby improving soil fertility [27]. Other systems such as check dams, earthen canals, and flood irrigation efficiently manage water distribution, reduce soil erosion, and ensure water availability. In Ethiopia, conservation techniques such as planting hedges or vegetative barriers, no-till or reduced tillage farming, and fallowing are commonly adopted [28]. In the study area, soil bunds, stone-faced bunds, gabions, and other traditional systems have been implemented.

### 2.7 Evaluating the effectiveness of the IWM practices

The evaluation was primarily concentrated on the technical elements, particularly the design and layout, and the implemented soil and water conservation (SWC) structures. A comprehensive assessment of the kebeles located along the transect line was conducted to gauge the effectiveness of the primary soil conservation structures. Existing physical SWC structures within the research area were compared to the established standards mentioned by Ref. [29]. To assess the effectiveness of the existing soil water conservation structures, measurements were taken through the field, including the vertical and horizontal intervals, and the slope of the terrain [30]. Recommended vertical and horizontal interval values for bunds, aligned with effective soil depth and land slopes, were provided in Table 2, as indicated by Ref. [29].

**Table 2:** Recommended VI and HI of bunds.

Slope (%)	>75cm (depth)		(25–50) cm (depth)		(50–75) cm(depth)	
	VI	HI	VI	HI	VI	HI
3	1	33	–	–	–	–
4	1	25	–	–	–	–
5	1	20	0.7	15	0.5	10
6	1	17	0.7	12	0.6	10
7	1	14	0.8	12	0.7	10
8	1	12	0.8	10	0.7	9
9	1	11	0.9	10	0.8	9
10	1	10	0.9	9	0.8	8
11	1.1	10	1	9	0.9	8
12	1.1	9	1	8	0.9	8
13	1.2	9	1.1	8	1	8
14	1.2	8	1.1	8	1	7
15	1.2	8	1.1	7	1	7
16	1.3	8	1.1	7	1	6
17	1.3	8	1.2	7	1.1	6
18	1.3	7	1.2	7	1.1	6
19	1.3	7	1.2	6	1.1	6
20	1.4	7	1.2	6	1.1	6

The governance and institutional evaluation involved policy and regulatory assessment, institutional performance, and stakeholder analysis [31]. The policy and regulatory assessment focused on analyzing the effectiveness of policies and regulations that support IWM practices. This involved reviewing policy documents, conducting stakeholder interviews, and assessing enforcement and compliance levels. Institutional performance was evaluated by examining the capacity, coordination mechanisms, and resource availability of institutions responsible for watershed management. Integrated and cross-cutting methods were applied using GIS and Remote Sensing (Sentinel-2) to monitor land use changes and vegetation.

#### 2.7.1 LULC/ecological indicators

These indicators are measurable parameters used to assess and monitor the condition, LULC dynamics, and various aspects of ecosystems and landscapes [32]. They provide essential information for understanding how natural and human-induced changes impact the environment over time [33]. It provides valuable information about the condition and dynamics of LULC changes. Here are some key types of these indicators such as vegetation which measures the extent and density of plant cover in an area. Land use change, tracks changes in LULC over time, such as urban expansion, bare land, deforestation, and agricultural intensification.

#### 2.7.2 Institutional indicators

Institutional indicators are used to assess the effectiveness of governance structures, policy coherence, collaboration with stakeholders and the community, financial constraints, and monitoring of existing IWM practices [31,34]. These indicators offer perspectives on the performance of institutions in fulfilling their responsibilities, maintaining standards, working in collaboration with the community, and promoting watershed sustainability. It reflects the effectiveness, efficiency, and equity of services provided by public institutions.

### 2.8 Sampling technique and sample size

A random sampling technique was employed in the study. The population sample size was selected from the target population for both kebeles. It includes Kebeles agriculture office experts and potential farmers. The questionnaires were distributed to those samples to collect the relevant data like trends of LULC, causes, priority issues, and dominant SWC measures. The sample size was determined based on [35] using Eq. (4).

(4) $$n=\frac{N}{1+N\left[{\mathrm{e}}^{2}\right]}$$

Where, n = simple size, N = total population, e = margin of error 10 %.

### 2.9 Data analysis

The data analysis combined both qualitative and quantitative information to deliver a thorough evaluation. Geospatial analysis was conducted using GIS tools to map and analyze spatial data, identifying patterns and changes in land use over selected years. A SWOT (Strengths, Weaknesses, Opportunities, Threats) analysis was performed to identify the strengths and limitations of current practices and explore new opportunities. Thematic analysis was applied to qualitative data from interviews, focus group discussions, and open-ended survey responses to identify key themes and patterns. By employing these data analysis methods, the effectiveness of current IWM practices was comprehensively assessed, leading to the development of evidence-based, innovative strategies.

## 3 Results and discussions

### 3.1 FAO soil classification

**Fig. 3:**
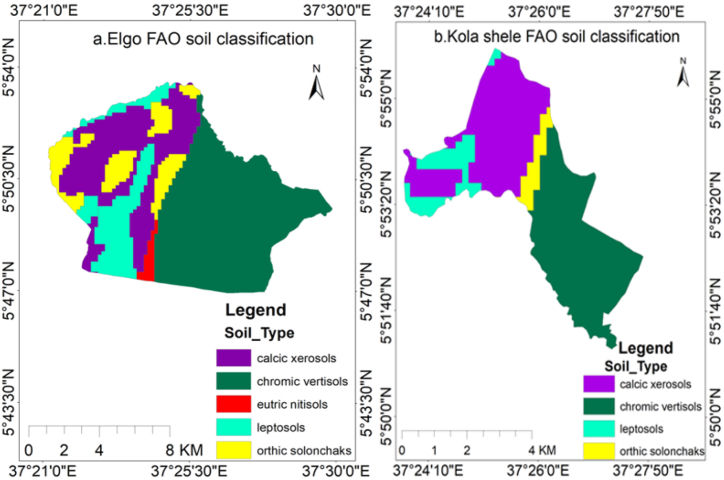
FAO soil classification for Elgo (a) and Kola Shele (b) kebeles.

According to the FAO soil classification performed with ArcGIS, the soil types found in Elgo and Kola Shele Kebele included chronic vertisols, calcic xerosols, leptosols, orthic solonchaks, and eutric nitisols, with chronic vertisols, calcic xerosols, leptosols, and orthic solonchaks being identified respectively. The soil descriptions are presented in Fig. 3 (a and b) as follows.

### 3.2 Field assessment of resources degradation

Based on the results, the researchers identified specific concerns during transect walks and discussions with local communities in both kebeles. These issues included deforestation, overgrazing, and encroachment by illegal farmers. The outcomes of these issues were noted to include the clearing of agricultural land, the formation of gullies, increased flooding in fields, displacement of downstream residents from their original areas, and sediment accumulation in canals.

**Fig. 4:**
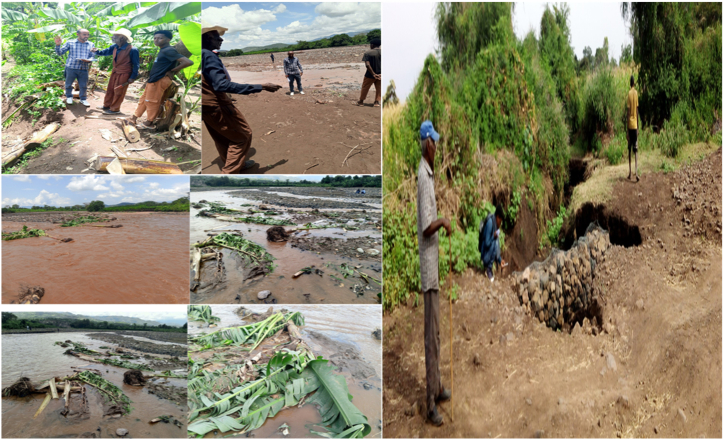
Elder man's discussion and observed problems for resource degradation.

Through interviews with farmers, it was disclosed that numerous individuals had encountered recurrent farmland loss owing to the emergence of nine new channels within the farmland of Elgo Kebele. Ato Egigu Ayalew mentioned that these gullies developed over five years due to the ongoing expansion of agricultural land by upstream farmers, combined with deforestation. Fig. 4 portrays an elderly man engaged in a conversation about land degradation and observed problems.

### 3.3 Topographic characteristics

**Fig. 5:**
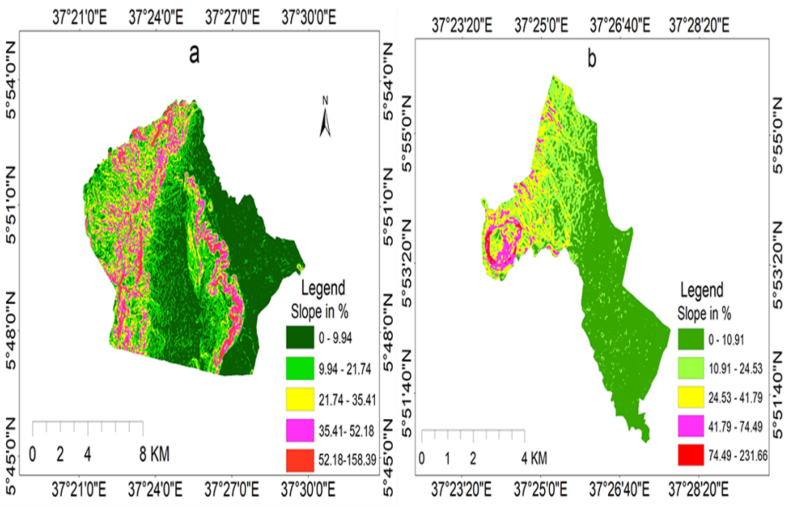
(a), (b) Slope map of Elgo and Kola Shele kebele, respectively.

Based on the results, five distinct slope categories were identified, as depicted in Fig. 5 (a and b). A small portion of the area was located on a steep slope greater than 50 %, while the majority of the region, specifically Elgo Keble, consisted of gentle slopes ranging from 0 to 10 %. Consequently, it can be inferred that the hillside contributes to significant soil erosion compared to the lower-lying regions.

### 3.4 Evaluating the effectiveness of IWM practices

#### 3.4.1 Constructional and design layout indicators

The study's slope characteristics ranged from hilly to flat, indicating that SWC practices would vary accordingly. Assessing the effectiveness of existing IWM practices helps to identify the constraints and provide a suggestion for improving IWM practices [30]. The design and layout of the actual IWM practices constructions were the focus of the evaluation. In the study area, bunds were widely used and dispersed practically across the sampling plots. The construction, and design layout performance evaluation of the existing IWM practices based on Minster of Agriculture standards was described in Table 3.

**Table 3:** Performance evaluation of the existing IWM practices based on MOA standards.

Plot 1	Slope (%)	HI(m)	Recommended	VI(m)	Recommended	Status
1	12	50	8–9	6	0.9-1.1	Above the standard
2	13	30	8–9	4	1–1.2	Above the standard
3	8	25	9–12	2	0.7–1	Above the standard
4	4.16	24	25–33	1	0.5–1	Standard
5	6.67	15	10–15	1	0.7–1	Standard
6	10	20	8–10	2	0.8–1	Above the standard
7	7.14	28	10–14	2	0.7–1	Above the standard
8	15	20	7–8	3	1–1.2	Above the standard
9	20	15	7–6	3	1.1–1.4	Above the standard
10	5	20	10–20	1	0.5–1	Standard
11	13.3	15	8–9	2	1–1.2	Above the standard
12	20.8	24	6–10	5	1–1.5	Above the standard
13	20	15	7–6	3	1.1–1.4	Above the standard
14	6.67	30	10–14	2	0.7–1	Above the standard
15	5.72	35	10–20	2	0.5–1	Above the standard
16	3.57	28	25–33	1	0.5–1	Standard
17	6.45	31	10–17	2	0.6–1	Above the standard
18	3.03	33	25–33	1	0.5–1	Standard
19	4.76	42	20–25	2	0.5–1	Above the standard
20	8	25	9–12	2	0.7–1	Above the standard
21	8.3	16	10–14	2	0.5–1	Above the standard
22	7.5	12	8–10	3	0.7–1	Above the standard
23	6	14	12–15	1	0.8–1	Standard
24	9.5	35	25–30	2	0.7–1	Above the standard
25	12.8	25	20–24	3	0.6–1	Above the standard
26	16.5	16	15–18	1	1–1.2	Standard
27	17	27	25–23	2	1.1–1.4	Above the standard
28	17.5	15	9–12	3	1–1.5	Above the standard

Among the 28 sapling points in the study, some of the applied IWM practices did not meet the design standards. According to Refs. [29,30] standard assessments, which considered factors like vertical and horizontal intervals as well as soil characteristics, a 25 % effectiveness rating for the implemented IWM practices in the study. Some bund structures were destroyed and silted up along the transect line observed as, a result of incorrect tillage between the bunds and runoff that topped the bunds. For the same slope and soil conditions, there was no typical bund design or layout.

The slope distribution map reveals that Elgo (0-73) % covers most of the land, with a slope range of (0-53) % for the vast majority of the land and more than 53 % for a small portion of the hillside. This demonstrates that the bund is insufficient to prevent soil loss brought on by topographic changes. According to Ref. [29], hillside terracing and revegetation are advised when the land slope is greater than 50 %. Integrated watershed management is required in the study area.

Scholars such as [30] assessed the IWM challenges, from 17 plots where IWM practices were constructed, only 20.58 % (bund gradient and vertical intervals) were designed based on the package specifications. The problem was serious mainly on bund spacing and vertical intervals. Creating awareness, capacity building and application of SWC practices based on the package specifications are the critical areas looked at in depth. Collaboration with relevant experts is crucial in planning, selecting appropriate SWC practices, and effectively implementing and maintaining soil conservation measures to positively impact community livelihoods. [36], identified several significant limitations in their assessment. These included inadequate community participation, non-alignment of structural designs with established standards, improper timing of implementation, insufficient variety in SWC measures, and neglect of regular maintenance and management of these practices. These factors were noted as major challenges affecting the effectiveness of the intervention. In the Maego watershed of northern Ethiopia, as reported by Ref. [37], various physical IWM practices were implemented across different land types including farmlands, closure areas, and grazing lands. These measures varied according to the slope gradient: on upper slopes, practices included shallow trenches with stone bunds, hillside terraces, and stone bunds; middle slopes featured hillside terraces, Halfmoon structures, and stone bunds; while lower slopes included deep trenches with stone bunds, gabion check dams, loose stone check dams, and percolation ponds. The placement and spacing of these IWM practices were determined by the gradient of the slope, with closer spacing employed on steeper slopes to effectively manage water runoff and erosion.

#### 3.4.2 The current status of IWM practices

The functionality of the existing practices was assessed during field investigation through the study area. Table 4 (a), (b) shows the number of existing practices percentage of functionality, and non-functionality of the existing IWM practices The area coverage of LULC classification for Elgo and Kola Shele kebele is presented in Table 5 for 2022 as shown below.

**Table 4:** The current status of IWM practices in Elgo (a) and Kola Shele kebeles (b) through field visiting.

No.	Types conservation practices	Elgo kebele				
		functional	Non-functional	Total	% of functionality	% of Non-functional
1	soil bund	10	35	45	22.22	77.78
2	Stone-faced bund	13	26	39	33.33	66.67
3	Gabion work	6	16	22	27.27	72.73

No.	Types conservation practices	Kola Shele kebele				
		functional	Non-functional	Total	% of functionality	% of Non-functional
1	soil bund	7	27	34	20.58	79.42
2	Stone-faced bund	8	20	28	28.57	64.29
3	Gabion work	4	9	13	30.77	69.23

**Table 5:** LULC description for 2022.

No	Lulc-type	Elgo		Kola Shele	
		Area (ha)	Area (%)	Area (ha)	Area (%)
1	Agriculture	5826.52	49.55	1079.32	48.92
2	Bare land	1587.10	13.50	306.35	13.89
3	Settlement	167.74	1.43	55.02	2.49
4	Forest	3830.23	32.58	698.13	31.65
5	Water Bodies	346.25	2.94	67.29	3.05

Based on the findings from field observation, the effectiveness of the soil bund, stone-faced bund, and gabion structures in Elgo kebele was observed to be 22.22 %, 33.33 %, and 27.27 % for functionality, and 77.78 %, 66.67 %, and 72.73 % for non-functionality, respectively. Whereas in Kola Shele kebele, the effectiveness of the soil bund, stone-faced bund, and gabion structures was found to be 20.58 %, 28.57 %, and 30.77 % for functionality, with non-functionality rates recorded at 79.42 %, 64.29 %, and 69.23 %, respectively. Elgo kebele demonstrates relatively better effectiveness in implementing IWM practices compared to Kola Shele kebele, primarily because Kola Shele kebele has steeper topographic characteristics.

According to Ref. [37] the effectiveness of IWM practices following project completion. Their assessment revealed a significant decline of 47–64 % in the condition of physical IWM practices once project support was withdrawn. The primary issues identified included inadequate periodic maintenance and limited support by biological conservation measures [38]. The findings underscore the substantial challenges faced by watershed technologies after project phases conclude. Therefore, the study advocates for collaborative efforts involving communities, governmental bodies, and non-governmental organizations to ensure sustainable resource management.

Following the phase-out of IWM projects, there was a noticeable decline in both the size and quality of all IWM practices. Specifically, damages ranged from 40 % (for hillside practices) to 70.5 % (for stone bunds) on upper slopes. Middle slope practices experienced damage between 38.7 % (for half-moons) and 65.9 % (for stone bunds). On lower slopes, reductions ranged from 32.5 % to 80.3 % (for gabion check dams). These findings align with [39], who reported a 40 % destruction rate of check dams two years after project phases ended in watersheds near Hagere Selam, northern Ethiopia. Key factors contributing to the failure of implemented IWM activities post-project phase-out included shortages of materials like gabions and cement, insufficient maintenance, overgrazing, and limited local capacity to sustain the implemented measures. The Goba District of southeast Ethiopia [40], reported a complete loss of stone bunds due to a lack of maintenance and overgrazing. The Wyebla Watershed in northwest Ethiopia [30], noted that 84.6 % of check dams were destroyed, mainly because of free grazing and inadequate maintenance.

Throughout these studies, common themes emerge regarding the challenges faced during IWM implementation and post-project phases. These include weak local institutions, insufficient community engagement, ineffective technology deployment, inadequate policy frameworks, limited stakeholder participation, and a lack of ownership [4,41].

#### 3.4.3 LULC/ecological indicators

**Fig. 6:**
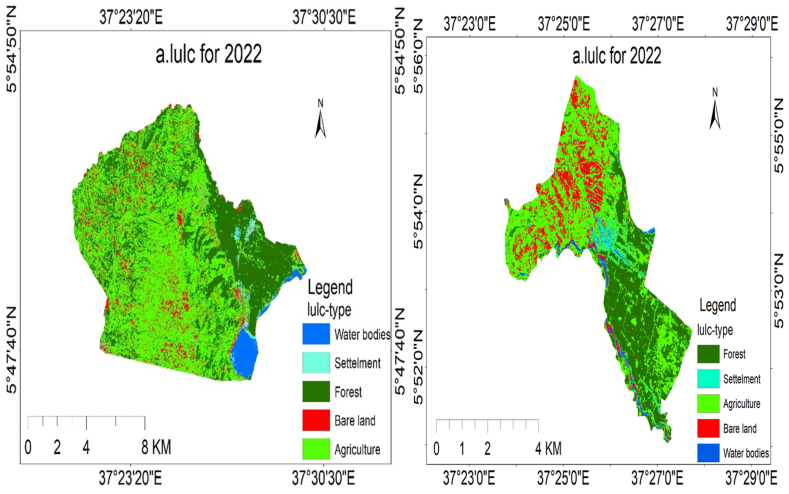
(a), (b) LULC classification for Elgo, and Kola Shele kebele for 2022, respectively.

**Fig. 7:**
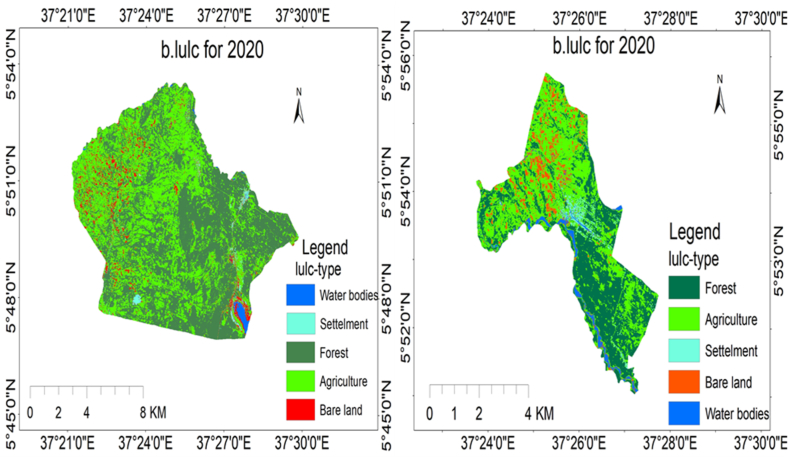
(a), (b) LULC classification for Elgo, and Kola Shele kebele for 2020, respectively.

**Fig. 8:**
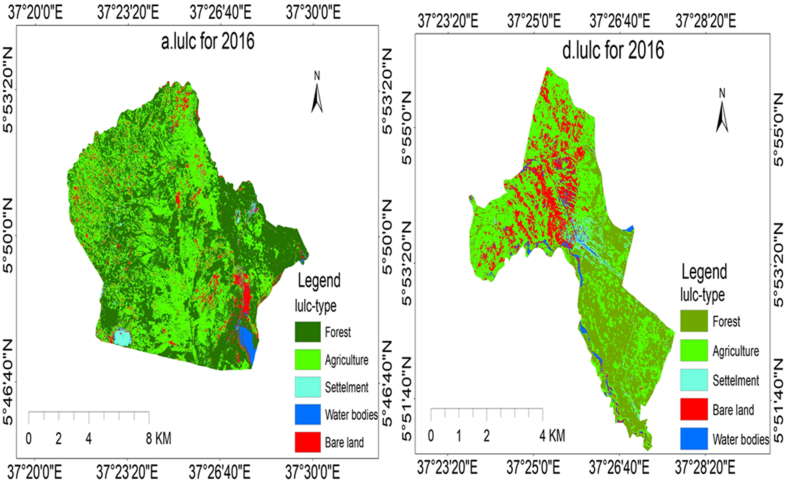
depicts the LULC classification for Elgo (a) and Kola Shele (d) kebele for 2016.

Through image classification, the researchers identified five types of land use and cover in Elgo and Kola Shele kebeles. The results of the classification process showed the existence of agricultural areas, bare land, settlements, forests, and water bodies. Each LULC classification and area coverage are described in Fig. 6, Fig. 7, Fig. 8 (a and b), and Table 5, Table 6, Table 7 as follows.

**Table 6:** LULC description for 2020.

No.	LULC-type	Elgo		Kola Shele		
		Area(ha)	Area (%)	Area(ha)	Area (%)	
1	Bare Land	596.89	5.08	163.86	7.43	
2	Agriculture	4829.21	41.07	1030.26	46.70	
3	Forest	6093.91	51.83	883.96	40.07	
4	Water	101.05	0.86	75.99	3.44	
5	Settlement	136.73	1.16	52.17	2.36

**Table 7:** LULC change from 2020 to 2022.

No.	LULC-type	LULC area change from (2022-2020)	
		Elgo	Kola Shele
1	Agriculture	1749.68	142.49
2	Bare land	526.13	2.85
3	Water	2127.12	49.06
4	Forest	−2660.53	−8.70
5	Settlement	1756.41	−185.76

In 2022, the LULC attributes of Elgo Kebele have been identified to encompass various classifications, including agriculture, arid land, water features, woodlands, and human settlements, constituting 55.51 %, 7.54 %, 2.94 %, 32.58 %, and 1.43 % of the overall area, respectively. In 2020, the LULC features of Elgo Kebele were categorized as follows: agriculture at 41.07 %, bare land at 5.08 %, water bodies at 0.86 %, forests at 51.83 %, and settlements at 1.16 %. The changes in LULC between 2020 and 2022, agricultural areas, bare land, water bodies, and settlements all experienced an increase of 14.44 %, 2.47 %, 2.09 %, and 0.26 % respectively. Conversely, there was a 19.25 % decrease in forested areas. Likewise, concerning Kola Shele kebele, the LULC examination demonstrates that from 2020 to 2022, there was a rise of 2.22 % in agricultural areas, 6.46 % in bare land, and 0.13 % in settlements. Conversely, there was a decline of 8.42 % in forested areas and 0.39 % in water bodies. These results unveil a significant deforestation trend and an increase in agricultural pursuits, expansion of barren land, and scattered settlement development in Elgo Kebele. The area coverage of LULC classification for Elgo and Kola Shele kebele for 2016 is shown in Table 8 below.

**Table 8:** LULC description for 2016.

No.	Lulc-type	Elgo		Kola Shele	
		Area(ha)	Area (%)	Area(ha)	Area (%)
1	Forest	5634.25	47.92	699.24	31.69
2	Agriculture	5237.68	44.27	1068.29	48.42
3	Bare land	645.51	5.49	346.48	15.70
4	Water	135.59	1.15	44.51	2.02
5	Settlement	104.67	0.89	47.68	2.16

**Fig. 9:**
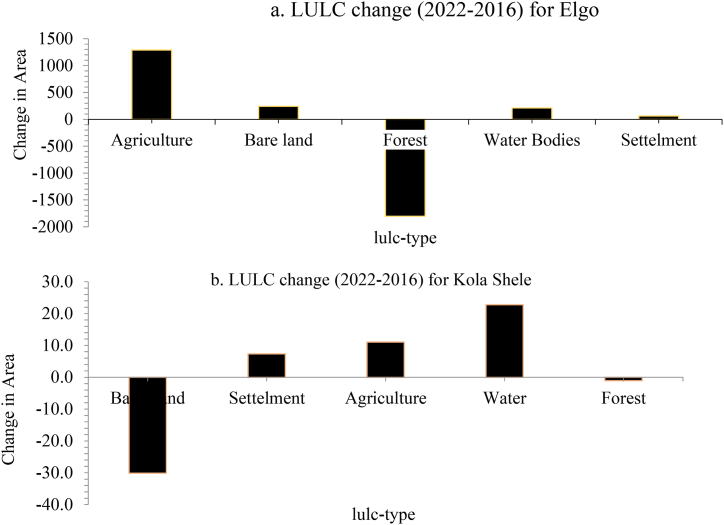
(a), (b) LULC change from 2022 to 2016 for Elgo and Kola Shele kebele respectively.

The LULC change between 2016 and 2022 was expressed in graph Fig. 9 (a and b) as follows.

Between 2016 and 2022, Elgo witnessed alterations in its LULC. The proportion of land allocated to agriculture, bare land, water bodies, and settlements rose by 11.24 %, 2.05 %, 1.79 %, and 0.54 %, respectively. Conversely, there was a decline of 15.34 % in forest area coverage during this timeframe as shown in Table 9.

**Table 9:** LULC change from 2016 to 2022.

No.	Lulc-type	Lulc area change from (2022-2016)	
		Elgo	Kola Shele
1	Agriculture	1288.84	11.03
2	Bare land	241.59	−40.13
3	Water	210.66	22.78
4	Forest	−1804.03	−1.04
5	Settlement	63.07	7.34

Several researchers have assessed LULC across various regions of Ethiopia, as illustrated in Ref. [42]. Their findings reveal a decline of 5.18 % in forested areas and 2.39 % in shrubland. Conversely, grassland, cropland, settlements, and water bodies have experienced an increase of 1.56 %, 6.18 %, 0.05 %, and 0.11 %, respectively. The substantial shifts in LULC may influenced by human activities in the region. According to Ref. [43], over 23 years, forests and grasslands have decreased by 8.5 % and 4.3 %, respectively, while agricultural and shrublands have expanded by 9.1 % and 3.7 %, respectively. These findings suggest that a significant portion of previously forested and grassland areas have been converted into rapidly expanding agricultural land, with deforestation driven primarily by farmland expansion.

As per research conducted by Ref. [44], it was found that from 1979 to 2017, the coverage of Forest, shrubland, Grassland, and water bodies declined by 59.3 %, 68.2 %, 32.7 %, and 5.1 %, respectively. Conversely, Urban built-up areas, cultivated land, and bare land witnessed an increase of 351 %, 105.3 %, and 41.9 %, respectively, over the same period. In another study by Ref. [45], it was observed that between 2020 and 2050, there was a notable rise of 6.36 % in cultivated land and 6.53 % in settlement areas. However, forestland and grassland decreased by 63.76 % and 22.325 %, respectively. Interviews with experts revealed that population growth and relocation were significant factors contributing to the expansion of agricultural land and reduction of forested areas. These findings carry crucial implications for decision-making processes and the formulation of land use policies aimed at enhancing land management strategies.

Similar to those findings, the present study suggests that notable shifts in land use and land cover are probably due to human activities, particularly the conversion of areas once covered by forests and grasslands into rapidly expanding agricultural lands, which emerge as the primary factors influencing the study area. Overall, the study observed frequent agroecological changes over time due to the outcomes of changes in LULC, which indirectly impacted the sustainability of IWM strategies.

##### 3.4.3.1 LULC accuracy assessment

There could be some errors in the classified LULC map. As a result, an accuracy assessment was conducted to determine whether the generated classification and what is found in reality are compatible. After evaluating the LULC, the accuracy assessment was checked and represented in Table 10 (a), and (b).

Based on the accuracy evaluation standards outlined in Table 10 for Elgo kebele, the most precisely identified land use and land cover was forest, succeeded by settlement, water bodies, bare land, and agriculture, in sequential order. Conversely, in Kola Shele kebele, as depicted in Table 6, forest and settlement were the most accurately classified land use and land cover, followed by water bodies, agriculture, and bare land, respectively (see Table 7).

**Table 10:** Accuracy assessments of LULC for Elgo (a) and Kola Shele(b) kebeles.

a. Elgo Kebele							
Accuracy assessment	Overall accuracy	Users Accuracy					Kappa coefficient (Ḱ)
		Forest	Agriculture	Bare land	Water	Settlement	
Values %	86	100	80	80	80	90	83
Remarks	Ok						
b. Kola Shele kebele							
Accuracy assessment	Overall accuracy	Users Accuracy					Kappa coefficient (Ḱ)
		Forest	Agriculture	Bare land	Water	Settlement	
Values %	88	100	80	70	90	100	85
Remarks	Ok						

In Elgo kebele, the overall accuracy and Kappa coefficient were 86 % and 83 % respectively. On the other hand, in Kola Shele kebele, the overall accuracy and Kappa coefficient were 88 % and 85 % respectively. Several studies had an accuracy assessment for example, researchers such as [21], reported an overall classification accuracy of 81.7 % and a kappa coefficient of 72.2 %. The kappa coefficient is rated as substantial and hence the classified image was found to be fit for further research. According to Ref. [46], the result shows that the overall accuracy of land use and land cover for 2014 is 82.00 % and Kappa is 77.02 % which is acceptable in both accuracy overall and Kappa accuracy. When comparing these results and threshold values, it can be noted that in the current study, the overall accuracy and Kappa coefficient values exceed the specified threshold for overall accuracy in both kebeles. Therefore, the level of agreement and percentage of reliability data of Kappa are perfect.

#### 3.4.4 Institutional indicators

According to the findings from the survey participants, the graphs below depict the outcomes derived from respondent input and show the level of the institutional indicators on IWM practices such as the effectiveness of governance structures, funding constraints, policy coherence, stakeholder collaboration with the community, and corruption in the study.

**Fig. 10:**
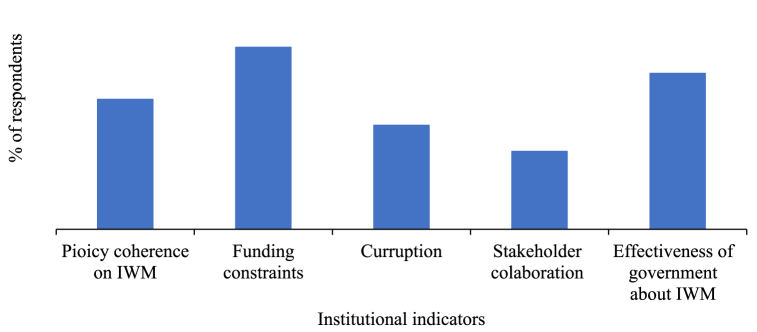
Institutional indicators for IWM strategies.

According to the answers provided by the respondents concerning in Fig. 10, the percentage order of policy coherence about watershed management, financial allocation for IWM strategies, corruption, stakeholder collaboration with the community, and the effectiveness of the government structures about IWM strategies were,20 %,28 %,16 %,12 %, and 24 % respectively. Based on the findings the most financial allocation for IWM strategies plays a significant role in sustainable watershed management strategies, stakeholder collaboration with the community exhibited comparatively less impact on watershed management [47].

### 3.5 Proposed alternative management strategies

From the investigation of the study area, under those kebeles, there are scorching issues at the upstream formation of the gully, deforestation, land sliding, and land degradation. In the downstream, there was the formation of excessive floods, loss of farmlands, and displacements of society from their homes. This is due to illegal expansion of plows, improper implementation of conservation structures, overgrazing, deforestation, less awareness creation about IWM in the community, no regulation for illegal farmers, and less monitoring of the implemented IWM. For Elgo and Kola Shell kebeles based on information gained from the agricultural office, farmers, and society the major constraints for the implemented integrated watershed management are major constraints for the present including reconciling the needs of resource-based planning with “people-first” objectives, the weak national research systems in developing countries, and local government/community commitment and the political will to allocate appropriate staff.

[29], proposed the required and appropriate conservation measurements based on slope, soil, and formation of lands. For cultivated land alley crops, bench terracing, broad and furrow, conservation tillage, gridded bund, graded Fanya juu, grass strip, and mulch are the main conservation measurements. The cultivated land having a slope of less than 15 % and vertisol, broad bed, and furrow were recommended. The **hillside terracing and revegetation are advised when the land slopes exceed 50 %** [29,48]. The suitable recommended conservation measures based on the soil type, slopes, and LULC type are described in Table 11.

**Table 11:** Recommended SWC structures.

Land use feature	Description	Recommended conservation practice
Cultivated land	Slopes of less than 15 % and soil: Heavy soils, e.g., Vertisol	Broaded furrow
	Slopes greater than 15 % and diverse soil compositions, when combined with contour structures.	Conservation tillages
	3%–50 % of slope, with all soil in wet, clayey soils in agricultural areas with high moisture levels	Graded bund
	3%–50 %, more on steeper slopes, with deep soil, in wet, deep clay soils in moist agroecological zones	Graded Fanya Juu
	Slopes with gradients of less than 15 % and all types of soil	Grass strip
Grassland	On a mild slope, the rangeland is adequately vegetated and the soil, except for severely damaged areas, is in good condition.	Controlled grazing
	After the grass is established, all slopes and all soil except heavily degraded	Cut and carry
	all slope and soil	Grassland improvement
Forest land	50 % - 100 % slope and heavily degraded land soil type	Hillside terracing
	all slope and soil except highly degraded land	Tree planting
	all slope and soil type	trench

The recommended techniques were proposed to protect the land degradation, however, especially for Elgo kebele, different formation gullies were formed. To recover the problems the present study proposes rehabilitation measurements for gullies formation in Table 12.

**Table 12:** Recommended SWC structures for gully rehabilitation.

Land use type	Description	Recommended measurements
Common to all lands	Slope Range: All and Soil Range: All	Area closure
	Slope Range: All and Soil Range: Take care of deeply weathered rock or loosely accumulated deposits	Check dam
	slope Range: 3–50 % and Soil Range: All	Cutoff drain
	Slope Range: 0–30 %, Soil Range: All, but take care of deeply weathered soils or loosely accumulated deposits	Gully rehabilitation
	Slope Range: All and Soil Range: All	Revegetation
	Slope Range: All (note specific limitation), Soil Range: All	Water harvesting
	Slope Range: 3–50 % Soil Range: All, but take care of deeply Weathered subsoils	Waterway

### 3.6 Community perceptions regarding IWM practices

IWM is a holistic approach that aims to manage the resources within a watershed to achieve the sustainability of the environment and community. Understanding community perceptions is crucial for the success of IWM practices, as community involvement and support can greatly influence the effectiveness and sustainability of these practices.

Using a random sampling technique 200 target populations were selected for both kebeles. The respondents include Kebele agriculture office experts, potential farmers, and the societies. The respondent highlighted that SWC practices have been in operation for two decades, yet issues related to soil erosion, sedimentation, and the formation of gullies persist without adequate resolution. The community's willingness to adopt watershed-based SWC practices is only moderate. The relevant authorities are fully cognizant of the existing challenges and propose measures to address the expansion of gullies along the Sego River, with regulations targeting illegal farming activities. The respondents emphasized the importance of constructing effective SWC structures and maintaining ongoing monitoring efforts. They stressed the significance of training and knowledge transfer from experienced kebeles and woredas, especially in handling various SWC structures. The respondents anticipate a continued trend of soil erosion and gully formation, leading to unmanageable issues for agriculture, with severe degradation of natural resources.

According to the findings from the survey participants, the graphs below depict the outcomes derived from respondent input, showcasing the patterns of LULC Change, the factors contributing to LULC change, the primary concerns regarding degraded land, and the prevailing conservation measures in the study area.

**Fig. 11:**
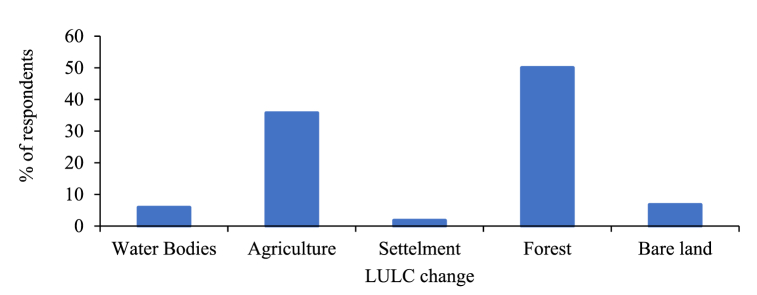
Trend of LULC change from (2016–2022).

According to the feedback received from the respondents regarding the progression of LULC change in Fig. 11 indicated percentages for the forest, agriculture, bare land, water bodies, and settlements as 49.99 %, 35.72 %, 6.69 %, 5.83 %, and 1.74 %, respectively. This suggests that the most substantial changes in LULC occurred in forests, while settlements experienced relatively minor changes during the study period in the designated area.

**Fig. 12:**
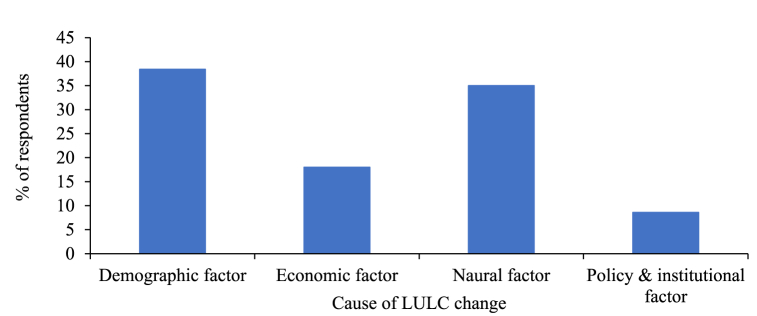
Cause of LULC changes.

According to the answers provided by the respondents concerning the reasons for LULC change, their respective proportions were demographic factors at 38.4 %, natural factors at 35 %, economic factors at 18 %, and policy and institutional factors at 8.6 % as shown in Fig. 12. This indicates that demographic factors played a significant role as the primary cause of LULC change, while policy and institutional factors exhibited comparatively minor changes in the designated area.

**Fig. 13:**
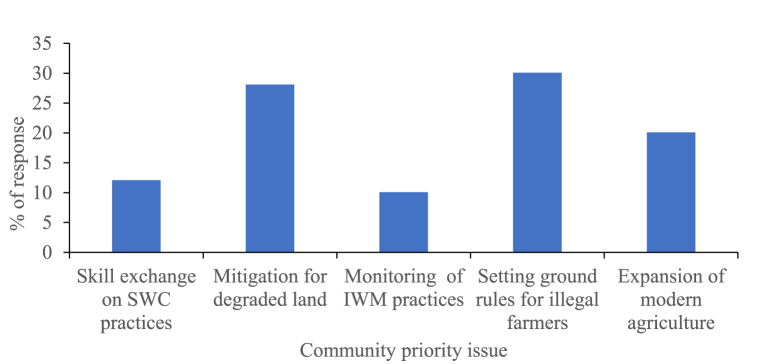
Priority issue.

According to the feedback from the respondents in Fig. 13, the most pressing concerns for degraded land were outlined as follows: setting regulations for illegal farmers at 30 %, mitigating degraded land at 28 %, expanding modern agriculture at 20 %, providing training and skill exchange on SWC practices at 12 %, and monitoring Integrated Irrigation Water Management practices at 10 %, respectively. This indicates that setting regulations for illegal farmers played a significant role in the degraded land while monitoring Integrated Irrigation Water Management practices exhibited comparatively minor changes in the study area.

**Fig. 14:**
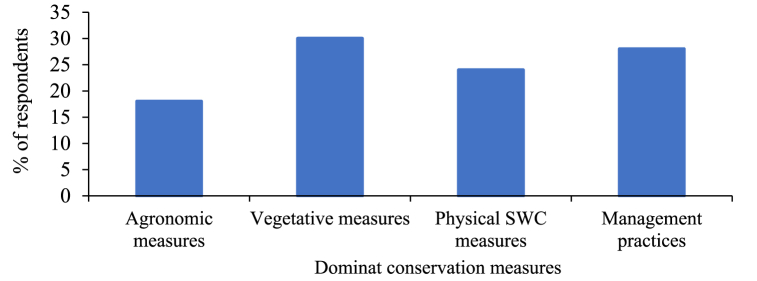
Dominant conservation measures.

Based on the data supplied by the respondents in Fig. 14, the predominant conservation measures comprised vegetative measures at 30 %, management practices at 28 %, physical SWC measures at 24 %, and agronomic measures at 18 %. These findings indicate that vegetative measures were the primary soil conservation methods, with agronomic measures demonstrating comparatively modest impact in the study area.

## 4 Conclusion

This study aimed to assess the effectiveness of IWM practices and propose new strategies to enhance watershed management using GIS and remote sensing (sentinel-2). The LULC changes in Elgo Kebele, significant changes in LULC were observed between 2016 and 2022, with agriculture expanding by 11.24 %, while forests experienced a notable decline of 15.34 %. In Kola Shele, LULC changes were subtler, with minor increases in agriculture (0.5 %), water bodies (1.03 %), settlement (0.033 %), and slight decreases in bare land (1.82 %), and forest (0.05 %). GIS and remote sensing data from Sentinel-2 proved invaluable for analyzing LULC changes and assessing the effectiveness of SWC practices. These technologies offer precise and up-to-date information essential for informed decision-making in watershed management. The effectiveness of SWC systems analysis revealed that only 25 % of the sampled plots met the MOA's criteria for vertical and horizontal intervals. This indicates significant challenges in the performance and implementation of existing SWCS. The effectiveness of the implemented IWM practices is relatively poor performing scale. Essential conservation practices for cultivated land were identified, including broad and furrow methods, conservation tillage, grass strips, and mulching. Gabion measurements were taken to prevent the farmland's gullies from getting larger. The research highlights significant land use changes in Elgo and Kola Shele kebeles, identifies gaps in current soil water conservation practices, and proposes targeted, site-specific conservation strategies. Comprehensive field observations, surveys, and stakeholder consultations provided a holistic understanding of the current state of watershed management practices. Engaging local communities and stakeholders is crucial for the successful implementation and sustainability of IWM strategies.

The sustainable management and future outlook are the adoption of the proposed strategies has the potential to improve watershed management, ensuring the sustainable use of water and land resources. Continuous monitoring and adaptive management practices will be essential to address emerging challenges and ensure long-term success.

## Data availability Statement

All relevant data used in this study have been provided and collected from various sources.

## CRediT authorship contribution statement

**Amare Tadesse Muche:** Writing – review & editing, Writing – original draft, Visualization, Validation, Software, Formal analysis, Conceptualization. **Yohannes Smeneh Ketsela:** Writing – original draft, Visualization, Software, Formal analysis. **Belete Meketaw Ali:** Writing – review & editing, Methodology, Formal analysis.

## Declaration of competing interest

The authors declare the following financial interests/personal relationships which may be considered as potential competing interests:Amare Tadesse reports financial support, administrative support, equipment, drugs, or supplies, statistical analysis, and travel were provided by Arba Minch University. Amare Tadesse Muche reports a relationship with Arba Minch University that includes: employment. Amare Tadesse Muche has a patent licensed to Yes. There is no conflicting interest among the authors. If there are other authors, they declare that they have no known competing financial interests or personal relationships that could have appeared to influence the work reported in this paper.
