# Supplementary material for: Assessing the effectiveness of integrated watershed management practices and suggesting innovative strategies in southern Ethiopia
Source: Heliyon. 2024 Sep 28;10(19):e38619. doi: 10.1016/j.heliyon.2024.e38619 (PMC11483310; doi:10.1016/j.heliyon.2024.e38619)
Supplement: Multimedia component 1 [file mmc1.docx]

**Appendix A. Supplementary data**

**A1 Soil Parameter’s results**

*A1.1 Soil texture*

It is the relative portion of the various size groups of individual soil grains mainly the percentage of (sand, silt clay) in the mass of soil. The United States Department of Agriculture defines twelve major soil texture classifications (sand, loamy sand, sandy loam, loam, silt loam, silt, sandy clay loam, clay loam, silty clay loam, sandy clay, silty clay, and clay). Soil textures are determined by the fractions of sand, silt, and clay in the soil. Therefore, using USDA classification of the soil type for this study is determined as shown in Tables (a), and (b) as follows.

Table 1 (a), (b) Soil texture analysis for Kola shele and Elgo kebele respectively

| 1. Kola Shele soil classification | | | | | |
| --- | --- | --- | --- | --- | --- |
| Sample point | Sample Name | % Clay | % Silt | % Sand | Soil texture |
| Point 1 | KL1(0-10cm) | 30 | 20 | 50 | sandy clay loam |
|  | KL2(10-20cm) | 20 | 28 | 52 | sandy loam |
|  | KL3(20-30cm) | 19 | 25 | 56 | sandy loam |
| Point 2 | KL1(0-10cm) | 17 | 28 | 55 | sandy loam |
|  | KL2(10-20cm) | 20 | 18 | 53 | sandy loam |
|  | KL3(20-30cm) | 22 | 21 | 51 | sandy clay loam |
| Point 3 | KL1(0-10cm) | 26 | 4 | 70 | sandy clay loam |
|  | KL2(10-20cm) | 24 | 14 | 62 | sandy clay loam |
|  | KL3(20-30cm) | 36 | 4 | 60 | sandy loam |
| Point 4 | KL1(0-10cm) | 20 | 30 | 50 | loam |
|  | KL2(10-20cm) | 6 | 64 | 30 | sandy loam |
|  | KL3(20-30cm) | 28 | 52 | 20 | sandy clay loam |
| Point 5 | KL1(0-10cm) | 16 | 16 | 68 | sandy loam |
|  | KL2(10-20cm) | 12 | 40 | 48 | sandy loam |
|  | KL3(20-30cm) | 10 | 64 | 26 | sandy loam |
| Point 6 | KL1(0-10cm) | 21 | 47 | 32 | loam |
|  | KL2(10-20cm) | 22 | 44 | 34 | loam |
|  | KL3(20-30cm) | 24 | 38 | 38 | loam |
| Point 7 | KL1(0-10cm) | 22 | 52 | 26 | silty loam |
|  | KL2(10-20cm) | 26 | 53 | 21 | silty loam |
|  | KL3(20-30cm) | 28 | 53 | 19 | silty clay loam |
| Point 8 | KL1(0-10cm) | 32 | 23 | 45 | sandy clay loam |
|  | KL2(10-20cm) | 30 | 23 | 47 | sandy clay loam |
|  | KL3(20-30cm) | 30 | 20 | 50 | sandy clay loam |
| Point 9 | KL1(0-10cm) | 17 | 30 | 53 | sandy loam |
|  | KL2(10-20cm) | 18 | 28.6 | 53.4 | sandy loam |
|  | KL3(20-30cm) | 19.5 | 27.7 | 52.8 | sandy loam |
| Point 10 | KL1(0-10cm) | 16 | 31.7 | 52.3 | sandy loam |
|  | KL2(10-20cm) | 18 | 32 | 50 | sandy loam |
|  | KL3(20-30cm) | 19 | 30 | 51 | sandy loam |
| Point 11 | KL1(0-10cm) | 18.5 | 26.5 | 55 | sandy loam |
|  | KL2(10-20cm) | 19 | 29 | 52 | sandy loam |
|  | KL3(20-30cm) | 17 | 29 | 54 | sandy loam |
| Point 12 | KL1(0-10cm) | 15 | 34 | 51 | sandy loam |
|  | KL2(10-20cm) | 18 | 29.8 | 52.2 | sandy loam |
|  | KL3(20-30cm) | 16.2 | 32.5 | 51.3 | sandy loam |
| Point 13 | KL1(0-10cm) | 18.7 | 27.9 | 53.4 | sandy loam |
|  | KL2(10-20cm) | 35 | 19 | 46 | sandy clay loam |
|  | KL3(20-30cm) | 31 | 24 | 45 | sandy clay loam |
| Point 14 | KL1(0-10cm) | 32 | 32.2 | 35.8 | clay loam |
|  | KL2(10-20cm) | 31.8 | 38.2 | 30 | clay loam |
|  | KL3(20-30cm) | 28 | 42 | 30 | clay loam |
| Point 15 | KL1(0-10cm) | 26 | 45 | 29 | loam |
|  | KL2(10-20cm) | 28 | 48 | 24 | loam |
|  | KL3(20-30cm) | 25 | 49 | 26 | loam |
| Point 16 | KL1(0-10cm) | 20 | 31 | 49 | loam |
|  | KL2(10-20cm) | 21 | 31 | 48 | loam |
|  | KL3(20-30 cm) | 22.5 | 31.4 | 46.1 | loam |
| **average** |  | **22.6** | **32** | **44.4** | **Loam** |

| 1. Elgo soil classification |
| --- |

| Sample points | Sample Name | % Clay | % Silt | % Sand | Soil texture |
| --- | --- | --- | --- | --- | --- |
| Point 1 | EL1(0-10cm) | 20 | 20 | 60 | sandy loam |
|  | EL2(10-20cm) | 4 | 26 | 70 | sandy loam |
|  | EL3(20-30cm) | 20 | 20 | 60 | sandy loam |
| Point 2 | EL1(0-10cm) | 7 | 43 | 50 | sandy loam |
|  | EL2(10-20cm) | 18 | 18 | 64 | sandy loam |
|  | EL3(20-30cm) | 19 | 21 | 60 | sandy clay loam |
| Point 3 | EL1(0-10cm) | 26 | 4 | 70 | sandy clay loam |
|  | EL2(10-20cm) | 24 | 14 | 62 | sandy clay loam |
|  | EL3(20-30cm) | 36 | 4 | 60 | sandy loam |
| Point 4 | EL1(0-10cm) | 20 | 30 | 50 | loam |
|  | EL2(10-20cm) | 6 | 64 | 30 | sandy loam |
|  | EL3(20-30cm) | 28 | 52 | 20 | sandy clay loam |
| Point 5 | EL1(0-10cm) | 16 | 16 | 68 | sandy loam |
|  | EL2(10-20cm) | 12 | 40 | 48 | sandy loam |
|  | EL3(20-30cm) | 10 | 64 | 26 | sandy loam |
| Point 6 | EL1(0-10cm) | 21 | 47 | 32 | loam |
|  | EL2(10-20cm) | 22 | 44 | 34 | loam |
|  | EL3(20-30cm) | 24 | 38 | 38 | loam |
| Point 7 | EL1(0-10cm) | 22 | 52 | 26 | silty loam |
|  | EL2(10-20cm) | 26 | 53 | 21 | silty loam |
|  | EL3(20-30cm) | 28 | 53 | 19 | silty clay loam |
| Point 8 | EL1(0-10cm) | 32 | 23 | 45 | sandy clay loam |
|  | EL2(10-20cm) | 30 | 23 | 47 | sandy clay loam |
|  | EL3(20-30cm) | 30 | 20 | 50 | sandy clay loam |
| Point 9 | EL1(0-10cm) | 17 | 30 | 53 | sandy loam |
|  | EL2(10-20cm) | 18 | 28.6 | 53.4 | sandy loam |
|  | EL3(20-30cm) | 19.5 | 27.7 | 52.8 | sandy loam |
| Point 10 | EL1(0-10cm) | 16 | 31.7 | 52.3 | sandy loam |
|  | EL2(10-20cm) | 18 | 32 | 50 | sandy loam |
|  | EL3(20-30cm) | 19 | 30 | 51 | sandy loam |
| Point 11 | EL1(0-10cm) | 18.5 | 26.5 | 55 | sandy loam |
|  | EL2(10-20cm) | 19 | 29 | 52 | sandy loam |
|  | EL3(20-30cm) | 17 | 29 | 54 | sandy loam |
| Point 12 | EL1(0-10cm) | 15 | 34 | 51 | sandy loam |
|  | EL2(10-20cm) | 18 | 29.8 | 52.2 | sandy loam |
|  | EL3(20-30cm) | 16.2 | 32.5 | 51.3 | sandy loam |
| Point 13 | EL1(0-10cm) | 18.7 | 27.9 | 53.4 | sandy loam |
|  | EL2(10-20cm) | 35 | 19 | 46 | sandy clay loam |
|  | EL3(20-30cm) | 31 | 24 | 45 | sandy clay loam |
| Point 14 | EL1(0-10cm) | 32 | 32.2 | 35.8 | clay loam |
|  | EL2(10-20cm) | 31.8 | 38.2 | 30 | clay loam |
|  | EL3(20-30cm) | 28 | 42 | 30 | clay loam |
| Point 15 | EL1(0-10cm) | 26 | 45 | 29 | loam |
|  | EL2(10-20cm) | 28 | 48 | 24 | loam |
|  | EL3(20-30cm) | 25 | 49 | 26 | loam |
| Point 16 | EL1(0-10cm) | 20 | 31 | 49 | loam |
|  | EL2(10-20cm) | 21 | 31 | 48 | loam |
|  | EL3(20-30cm) | 23 | 32 | 45 | loam |
| Point 17 | EL1(0-10cm) | 30 | 24 | 46 | sandy clay loam |
|  | EL2(10-20cm) | 34 | 21 | 45 | sandy clay loam |
|  | EL3(20-30cm) | 31 | 23 | 46 | sandy clay loam |
| Point 18 | EL1(0-10cm) | 20 | 25 | 55 | sandy loam |
|  | EL2(10-20cm) | 19 | 29 | 52 | sandy loam |
|  | EL3(20-30cm) | 18 | 29 | 53 | sandy loam |
| Point 19 | EL1(0-10cm) | 32 | 31.8 | 36.2 | clay loam |
|  | EL2(10-20cm) | 35.5 | 29.5 | 35 | clay loam |
|  | EL3(20-30cm) | 34 | 30 | 36 | clay loam |
| Point 20 | EL1(0-10cm) | 25 | 39.8 | 35.2 | loam |
|  | EL2(10-20cm) | 28 | 34 | 38 | loam |
|  | EL3(20-30cm) | 27 | 36 | 37 | loam |
| Point 21 | EL1(0-10cm) | 30 | 31.5 | 38.5 | clay loam |
|  | EL2(10-20cm) | 31 | 33.7 | 35.3 | clay loam |
|  | EL3(20-30cm) | 29 | 31.3 | 39.7 | clay loam |
| Point 22 | EL1(0-10cm) | 18 | 27 | 55 | sandy loam |
|  | EL2(10-20cm) | 19 | 27 | 54 | sandy loam |
|  | EL3(20-30cm) | 20 | 28 | 52 | sandy loam |
| Point 23 | EL1(0-10cm) | 16 | 31 | 53 | sandy loam |
|  | EL2(10-20cm) | 18 | 27.8 | 54.2 | sandy loam |
|  | EL3(20-30cm) | 18.6 | 26.3 | 55.1 | sandy loam |
| **Average** |  | **23** | **32** | **46** | **Loam** |

**Appendix B**

Table 2 (c), (d) Erodibility analysis for Kola shele and Elgo kebele respectively

1. Kola shele

| Sample point | Sample Name | Soil texture | Erodibility (K) | |
| --- | --- | --- | --- | --- |
| Point 1 | KL1(0-10cm) | sandy clay loam | 0.1512 |  |
|  | KL2(10-20cm) | sandy loam | 0.1676 |  |
|  | KL3(20-30cm) | sandy loam | 0.1676 |  |
| Point 2 | KL1(0-10cm) | sandy loam | 0.1704 |  |
|  | KL2(10-20cm) | sandy loam | 0.1582 |  |
|  | KL3(20-30cm) | sandy clay loam | 0.1599 |  |
| Point 3 | KL1(0-10cm) | sandy clay loam | 0.1024 |  |
|  | KL2(10-20cm) | sandy clay loam | 0.1446 |  |
|  | KL3(20-30cm) | sandy loam | 0.0980 |  |
| Point 4 | KL1(0-10cm) | Loam | 0.1690 |  |
|  | KL2(10-20cm) | sandy loam | 0.1921 |  |
|  | KL3(20-30cm) | sandy clay loam | 0.1743 |  |
| Point 5 | KL1(0-10cm) | sandy loam | 0.1551 |  |
|  | KL2(10-20cm) | sandy loam | 0.1844 |  |
|  | KL3(20-30cm) | sandy loam | 0.1902 |  |
| Point 6 | KL1(0-10cm) | Loam | 0.1783 |  |
|  | KL2(10-20cm) | Loam | 0.1762 |  |
|  | KL3(20-30cm) | Loam | 0.1716 |  |
| Point 7 | KL1(0-10cm) | silty loam | 0.1760 |  |
|  | KL2(10-20cm) | silty loam | 0.1778 |  |
|  | KL3(20-30cm) | silty clay loam | 0.1765 |  |
| Point 8 | KL1(0-10cm) | sandy clay loam | 0.1536 |  |
|  | KL2(10-20cm) | sandy clay loam | 0.1534 |  |
|  | KL3(20-30cm) | sandy clay loam | 0.1480 |  |
| Point 9 | KL1(0-10cm) | sandy loam | 0.1739 |  |
|  | KL2(10-20cm) | sandy loam | 0.1721 |  |
|  | KL3(20-30cm) | sandy loam | 0.1699 |  |
| Point 10 | KL1(0-10cm) | sandy loam | 0.1763 |  |
|  | KL2(10-20cm) | sandy loam | 0.1745 |  |
|  | KL3(20-30cm) | sandy loam | 0.1721 |  |
| Point 11 | KL1(0-10cm) | sandy loam | 0.1694 |  |
|  | KL2(10-20cm) | sandy loam | 0.1712 |  |
|  | KL3(20-30cm) | sandy loam | 0.1734 |  |
| Point 12 | KL1(0-10cm) | sandy loam | 0.1783 |  |
|  | KL2(10-20cm) | sandy loam | 0.1720 |  |
|  | KL3(20-30cm) | sandy loam | 0.1765 |  |
| Point 13 | KL1(0-10cm) | sandy loam | 0.1709 |  |
|  | KL2(10-20cm) | sandy clay loam | 0.1453 |  |
|  | KL3(20-30cm) | sandy clay loam | 0.1530 |  |
| Point 14 | KL1(0-10cm) | clay loam | 0.1618 |  |
|  | KL2(10-20cm) | clay loam | 0.1661 |  |
|  | KL3(20-30cm) | clay loam | 0.1712 |  |
| Point 15 | KL1(0-10cm) | Loam | 0.1723 |  |
|  | KL2(10-20cm) | Loam | 0.1739 |  |
|  | KL3(20-30cm) | Loam | 0.1753 |  |
| Point 16 | KL1(0-10cm) | Loam | 0.1708 |  |
|  | KL1(10-20cm) | Loam | 0.1687 |  |
|  | KL2(20-30cm) | Loam | 0.1705 |  |
| **Average** |  |  | **0.166** |  |

1. Elgo

| Sample point | Sample Name | Soil type | Erodibility(K) |
| --- | --- | --- | --- |
| Point 1 | EL1(0-10cm) | sandy loam | 0.1612 |
|  | EL2(10-20cm) | sandy loam | 0.1818 |
|  | EL3(20-30cm) | sandy loam | 0.1609 |
| Point 2 | EL1(0-10cm) | sandy loam | 0.1884 |
|  | EL2(10-20cm) | sandy loam | 0.1595 |
|  | EL3(20-30cm) | sandy clay loam | 0.1636 |
| Point 3 | EL1(0-10cm) | sandy clay loam | 0.1034 |
|  | EL2(10-20cm) | sandy clay loam | 0.1464 |
|  | EL3(20-30cm) | sandy loam | 0.0993 |
| Point 4 | EL1(0-10cm) | Loam | 0.1707 |
|  | EL2(10-20cm) | sandy loam | 0.1921 |
|  | EL3(20-30cm) | sandy clay loam | 0.1753 |
| Point 5 | EL1(0-10cm) | sandy loam | 0.1558 |
|  | EL2(10-20cm) | sandy loam | 0.1844 |
|  | EL3(20-30cm) | sandy loam | 0.1902 |
| Point 6 | EL1(0-10cm) | Loam | 0.1790 |
|  | EL2(10-20cm) | Loam | 0.1770 |
|  | EL3(20-30cm) | Loam | 0.1725 |
| Point 7 | EL1(0-10cm) | silty loam | 0.1798 |
|  | EL2(10-20cm) | silty loam | 0.1778 |
|  | EL3(20-30cm) | silty clay loam | 0.1765 |
| Point 8 | EL1(0-10cm) | sandy clay loam | 0.1538 |
|  | EL2(10-20cm) | sandy clay loam | 0.1555 |
|  | EL3(20-30cm) | sandy clay loam | 0.1517 |
| Point 9 | EL1(0-10cm) | sandy loam | 0.1743 |
|  | EL2(10-20cm) | sandy loam | 0.1721 |
|  | EL3(20-30cm) | sandy loam | 0.1699 |
| Point 10 | EL1(0-10cm) | sandy loam | 0.1763 |
|  | EL2(10-20cm) | sandy loam | 0.1745 |
|  | EL3(20-30cm) | sandy loam | 0.1721 |
| Point 11 | EL1(0-10cm) | sandy loam | 0.1694 |
|  | EL2(10-20cm) | sandy loam | 0.1712 |
|  | EL3(20-30cm) | sandy loam | 0.1734 |
| Point 12 | EL1(0-10cm) | sandy loam | 0.1790 |
|  | EL2(10-20cm) | sandy loam | 0.1720 |
|  | EL3(20-30cm) | sandy loam | 0.1765 |
| Point 13 | EL1(0-10cm) | sandy loam | 0.1709 |
|  | EL2(10-20cm) | sandy clay loam | 0.1453 |
|  | EL3(20-30cm) | sandy clay loam | 0.1557 |
| Point 14 | EL1(0-10cm) | clay loam | 0.1618 |
|  | EL2(10-20cm) | clay loam | 0.1661 |
|  | EL3(20-30cm) | clay loam | 0.1712 |
| Point 15 | EL1(0-10cm) | Loam | 0.1742 |
|  | EL2(10-20cm) | Loam | 0.1743 |
|  | EL3(20-30cm) | Loam | 0.1763 |
| Point 16 | EL1(0-10cm) | Loam | 0.1715 |
|  | EL2(10-20cm) | Loam | 0.1700 |
|  | EL3(20-30cm) | Loam | 0.1689 |
| Point 17 | EL1(0-10cm) | sandy clay loam | 0.1564 |
|  | EL2(10-20cm) | sandy clay loam | 0.1493 |
|  | EL3(20-30cm) | sandy clay loam | 0.1545 |
| Point 18 | EL1(0-10cm) | sandy loam | 0.1670 |
|  | EL2(10-20cm) | sandy loam | 0.1715 |
|  | EL3(20-30cm) | sandy loam | 0.1725 |
| Point 19 | EL1(0-10cm) | clay loam | 0.1618 |
|  | EL2(10-20cm) | clay loam | 0.1576 |
|  | EL3(20-30cm) | clay loam | 0.1588 |
| Point 20 | EL1(0-10cm) | Loam | 0.1723 |
|  | EL2(10-20cm) | Loam | 0.1666 |
|  | EL3(20-30cm) | Loam | 0.1686 |
| Point 21 | EL1(0-10cm) | clay loam | 0.1633 |
|  | EL2(10-20cm) | clay loam | 0.1641 |
|  | EL3(20-30cm) | clay loam | 0.1641 |
| Point 22 | EL1(0-10cm) | sandy loam | 0.1710 |
|  | EL2(10-20cm) | sandy loam | 0.1697 |
|  | EL3(20-30cm) | sandy loam | 0.1696 |
| Point 23 | EL1(0-10cm) | sandy loam | 0.1759 |
|  | EL2(10-20cm) | sandy loam | 0.1716 |
|  | EL3(20-30cm) | sandy loam | 0.1693 |
| **Average** |  | **0.167** | |

**Appendix C. Survey Questionnaires**

**Research Title**: Assessing the Effectiveness of Integrated Watershed Management Practices and Suggesting Innovative Strategies in Southern Ethiopia.

**The Specific Objectives of the Study**

- To evaluate the extent rate of land degradation,
- To evaluate the performance of existing IWM practices,
- To identify major gaps in existing IWM practices and formulate a new strategy.

**Dear respondent**

Thank you for participating in this survey aimed at evaluating the effectiveness of integrated watershed management practices and gathering insights to suggest innovative strategies. Your responses will contribute to enhancing water resource management efforts. Please answer the following questions thoughtfully and accurately.

**Section 1: Demographic Information**

1.1 Respondent’s name: -------------------------------------------------------

1.2 Gender:

- Male
- Female
- Other (please specify: ------------------------------------------------)

1.3 Age:

- Under 18
- 18-25
- 26-35
- 36-45
- 46-55
- 56 and above

1.4 Marital status (A) Married (B) Not married (C) Divorced/separated (D) Other, specify-----

1.5 Literacy / Educational Qualification:

- Did not join any formal schooling
- Adult education
- Writing and reading skills
- Elementary school (grades 1-8)
- High School
- TVET (H) Diploma complete
- University Degree
- Other (please specify: ------------------------------------------)

1.6 Occupation:

- Agriculture/Farming
- Environmental Conservationist
- Government Employee
- NGO/Non-profit Organization
- Researcher/Academician
- Other (please specify: ---------------------------------------------------)

1.7 Household size including yourself: A/ Male ---------------- B/ Female ------------

1.8 For how long have you lived in this village? -------------------------------------Year E.C

1.9 What are your major livelihood activities? List according to your priority

| No. | Livelihood activities | 1st | 2nd | 3rd | 4th | 5th | 6th |
| --- | --- | --- | --- | --- | --- | --- | --- |
| 1 | Agriculture |  |  |  |  |  |  |
| 2 | Livestock |  |  |  |  |  |  |
| 3 | Trade |  |  |  |  |  |  |
| 4 | Employment |  |  |  |  |  |  |
| 5 | Daily worker |  |  |  |  |  |  |
| 6 | Others |  |  |  |  |  |  |

1.10 How did you observe the trend of agricultural productivity in your life experience? A/ Increasing B/ Decreasing C/ Constant D/ Difficult to estimate

1.11 How you observed the trend of livestock production and productivity A/ Increasing B/ Decreasing C/ Constant D/ Difficult to estimate

**Section 2: Land ownership and LULC change characteristics**

2.1 How much is your total plot size/area in ha -----------------------------------------------------

2.2 Do you have more than one plot? A/Yes B/ No

2.3 Do you perceive land use and land cover change in the study area? A/ Yes B/ No C/ No idea

2.3.1 If yes how was the trend of the land use land cover change in their respective order?

| No. | Land use type | 1st | 2nd | 3rd | 4th | 5th | 6th |
| --- | --- | --- | --- | --- | --- | --- | --- |
| 1 | Agriculture |  |  |  |  |  |  |
| 2 | Forest |  |  |  |  |  |  |
| 3 | Bare land |  |  |  |  |  |  |
| 4 | Water bodies |  |  |  |  |  |  |
| 5 | Settlement |  |  |  |  |  |  |
| 6 | Others |  |  |  |  |  |  |

2.4 What are the causes of land use and land cover change in their respective order?

| No. | Drivers to LULCC | Yes | No |
| --- | --- | --- | --- |
| 1 | Demographic factors (population density, settlement) |  |  |
| 2 | Economic factors (agriculture, charcoaling, fuel wood, etc.) |  |  |
| 3 | Natural factors (high intensity of rainfall and drought) |  |  |
| 4 | Policy and institutional factors (soil and water conservation) |  |  |
| 5 | Others specify |  |  |

**Section 3: Communities' perceptions and attitude on the soil erosion problem**

3.1 Do you think there is a soil erosion problem in your locality/Kebele?

A/ Yes B/ No C/ No idea

3.2 What do you think are the causes of soil erosion in your farmland?

| No. | Cause of soil erosion | Agree | Disagree | Partially agree | Neutral |
| --- | --- | --- | --- | --- | --- |
| 1 | Topographic factors |  |  |  |  |
| 2 | Deforestation |  |  |  |  |
| 3 | High population growth and related factor |  |  |  |  |
| 4 | Expansion of agriculture |  |  |  |  |
| 5 | Nature of soil |  |  |  |  |
| 6 | Climatic factors |  |  |  |  |
| 7 | Other specify |  |  |  |  |

3.3 How are the trends of soil erosion over time on your farmlands? A/ Increasing B/ Decreasing C/ Stable D/ Difficult to estimate

3.3.1 If increasing, what are the dominant conservation measures in your Kebeles in their respective orders?

| No. | Conservation Measures | 1st | 2nd | 3rd | 4th | 5^th^ |
| --- | --- | --- | --- | --- | --- | --- |
| 1 | Agronomic measures (mulching, crop  rotation, strip cropping, and conservation  agriculture) |  |  |  |  |  |
| 2 | Vegetative measures (grass strip, orchard  strip, natural vegetation, and agroforestry) |  |  |  |  |  |
| 3 | Physical SWC measures (bunds, terracing, check dams, and water harvesting structures) |  |  |  |  |  |
| 4 | Management practices (fallowing of land,  composting, manuring, and grassing  management) |  |  |  |  |  |
| 5 | Other specify |  |  |  |  |  |

3.4 Did you support the implementation of SWC practices? A/ Strongly support B/Partially support C/ Not at all D/ Not decided

3.4.1 If you support it, why? ---------------------------------------------------------------------------

3.4.2 If you do not support it, why? -------------------------------------------------------------------

**Section 4: Perception and attitude of the people to the community-based participatory SWC practices**

4.1 How familiar are you with the concept of integrated watershed management?

- Very familiar
- Somewhat familiar
- Not familiar at all

4.2 Is their community-based participatory soil and water conservation (CBPSWC) in your Kebele? A/Yes B/ No C/ Not known

4.3 If ‘Yes’, when do CBPSWC practices begin in your locality? --------------------year

4.4 Have you participated in any integrated watershed management projects or activities before?

- Yes
- No

4.5 If yes, please briefly describe your involvement and the outcomes observed. ----------------

**Section 5: Current Practices Evaluation**

5.1 In your opinion, how effective are the current watershed management practices in your area?

- Highly effective
- Moderately effective
- Slightly effective
- Not effective at all

5.2 What are the main challenges or limitations you observe in the current watershed management practices? (Check all that apply)

- Demand for agriculture
- Lack of maintenance for the implemented SWC measures
- Lack of community participation
- Insufficient funding/resources
- Inadequate technical expertise in SWC practices
- Poor coordination among stakeholders
- Other (please specify)

**Section 6: Perception of the households to the priority issues in their locality**

6.1 What should be the priority issues that need the higher intervention of the government in the Kebele?

| No. | Community priority area | 1st | 2nd | 3rd | 4th | 5th | 6th |
| --- | --- | --- | --- | --- | --- | --- | --- |
| 1 | Training and skill exchange on SWC practices |  |  |  |  |  |  |
| 2 | Mitigation options for degraded land |  |  |  |  |  |  |
| 3 | Monitoring of the implemented IWM practices |  |  |  |  |  |  |
| 4 | Setting ground rules about illegal farmers |  |  |  |  |  |  |
| 5 | Expansion of modern agriculture |  |  |  |  |  |  |
| 6 | Specify others |  |  |  |  |  |  |

6.2 What do you want to say about the overall situation of SWC practices in your locality?..................................................................................................................................

**Section 7: Innovative Strategies**

7.1 In your view, what innovative approaches or technologies could enhance the effectiveness of integrated watershed management practices? Please describe briefly---------------------------

7.2 How can community participation and stakeholder engagement be improved to better support integrated watershed management efforts? Please describe briefly------------------------

7.3 Are there any specific policy recommendations or regulatory measures you believe would facilitate integrated watershed management?

**Section 8: Feedback and Suggestions**

8.1 Do you have any additional comments, feedback, or suggestions regarding integrated watershed management practices?

Thank you for your valuable participation in this survey. Your input will contribute significantly to advancing integrated watershed management efforts.

**Appendix D. Relevant images**


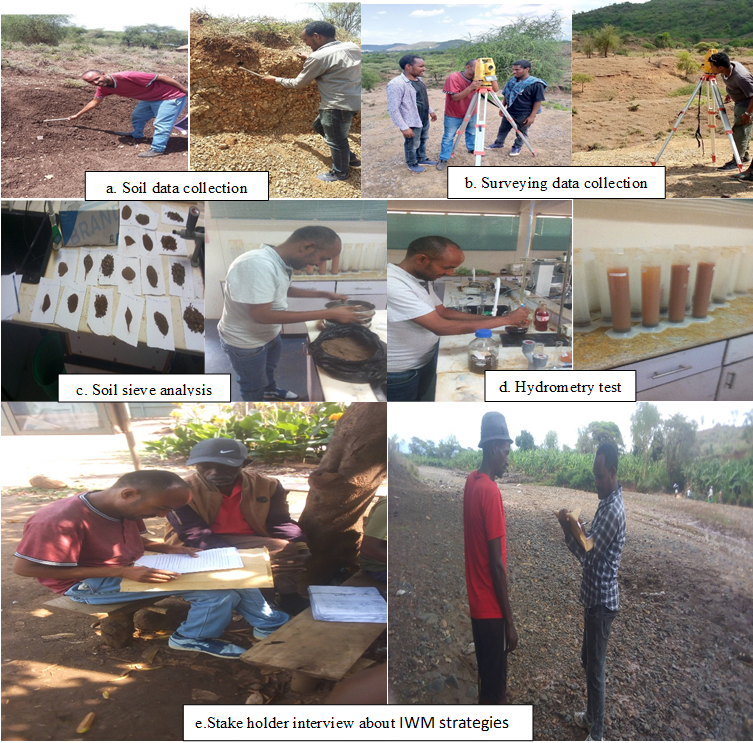


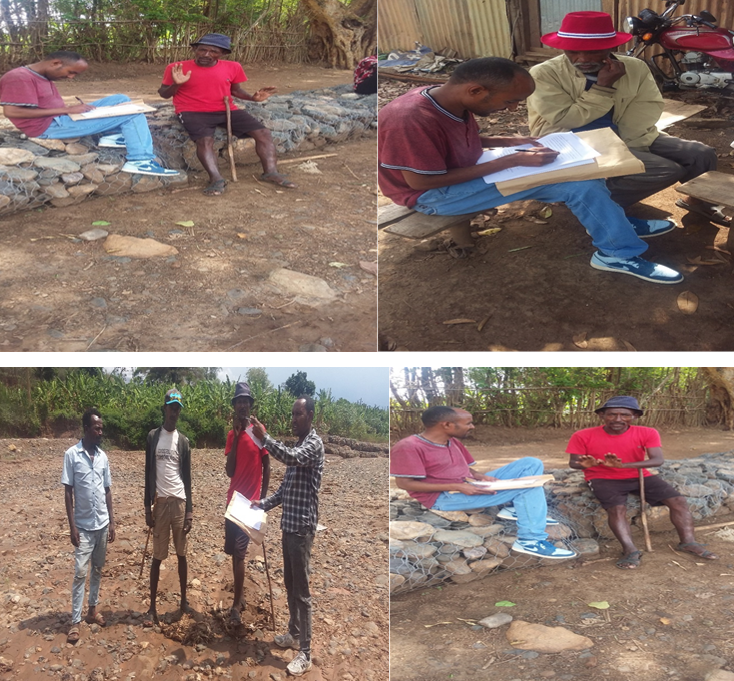


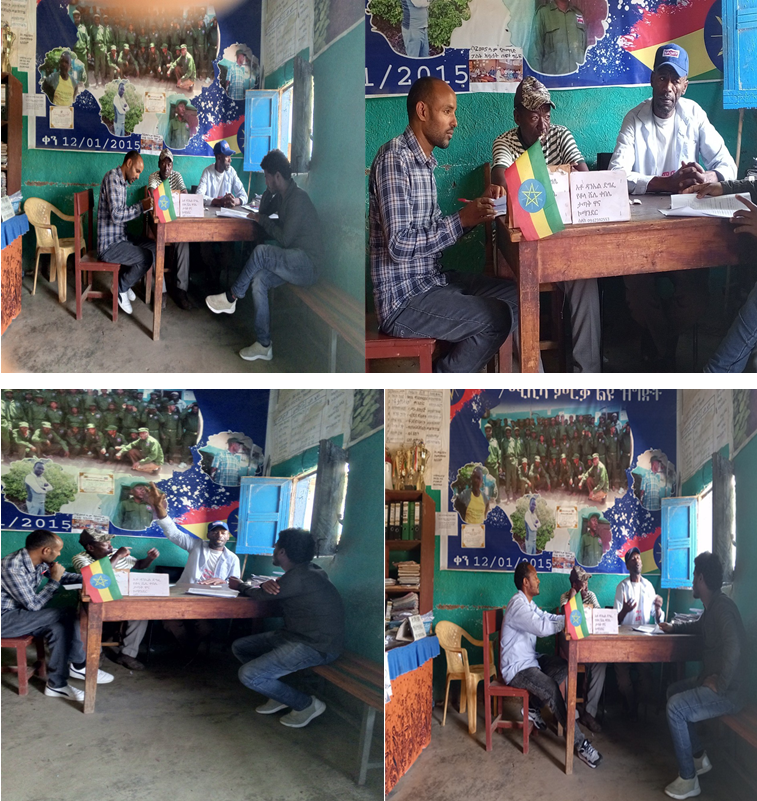

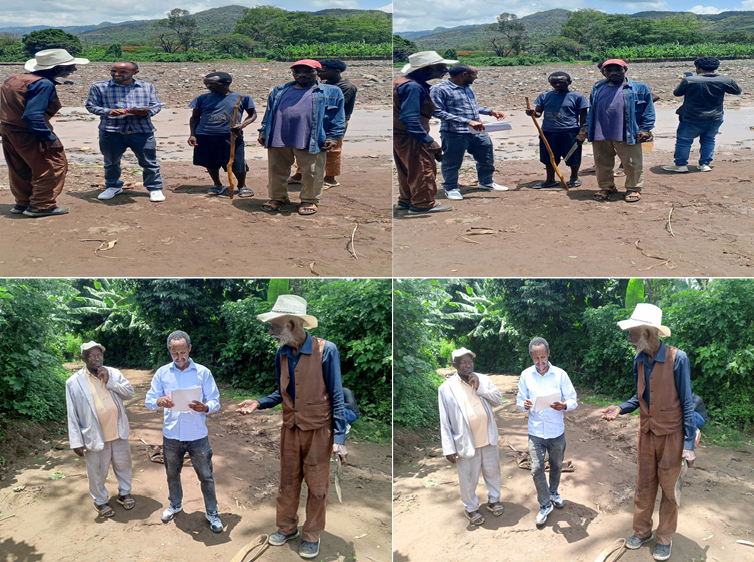


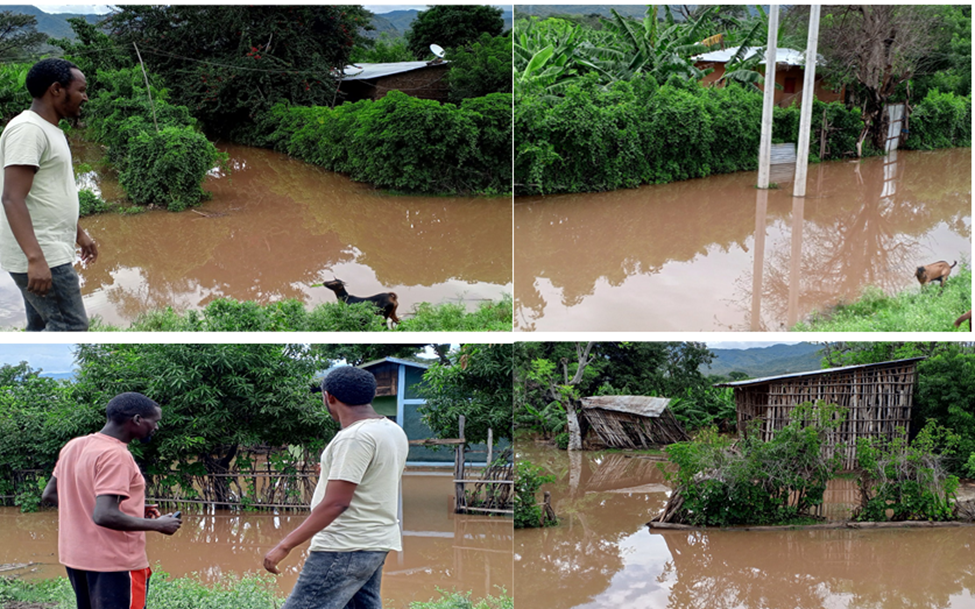


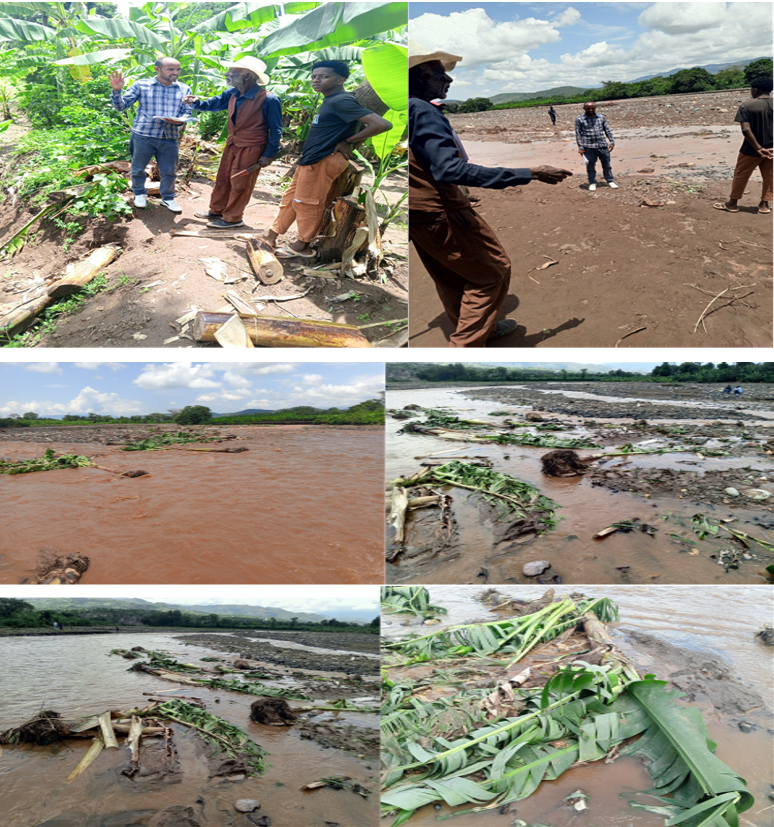


Fig. 1. Data collection, stakeholder consultation, and existing problems during field visiting
